# Insights on Molecular Mechanisms of Chondrocytes Death in Osteoarthritis

**DOI:** 10.3390/ijms17122146

**Published:** 2016-12-20

**Authors:** Edith Charlier, Biserka Relic, Céline Deroyer, Olivier Malaise, Sophie Neuville, Julie Collée, Michel G. Malaise, Dominique De Seny

**Affiliations:** Laboratory of Rheumatology, Groupe Interdisciplinaire de Génoprotéomique Appliquée (GIGA) Research, Centre Hospitalier Universitaire (CHU) de Liège, University of Liège, 4000 Liège, Belgium; biserka.relic@chu.ulg.ac.be (B.R.); celine.deroyer@ulg.ac.be (C.D.); olivier.malaise@chu.ulg.ac.be (O.M.); sophie.neuville@chu.ulg.ac.be (S.N.); julie.collee@student.ulg.ac.be (J.C.); michel.malaise@ulg.ac.be (M.G.M.); ddeseny@chu.ulg.ac.be (D.D.S.)

**Keywords:** osteoarthritis, apoptosis, necrosis, chondroptosis, autophagy, chondrocytes

## Abstract

Osteoarthritis (OA) is a joint pathology characterized by progressive cartilage degradation. Medical care is mainly based on alleviating pain symptoms. Compelling studies report the presence of empty lacunae and hypocellularity in cartilage with aging and OA progression, suggesting that chondrocyte cell death occurs and participates to OA development. However, the relative contribution of apoptosis per se in OA pathogenesis appears complex to evaluate. Indeed, depending on technical approaches, OA stages, cartilage layers, animal models, as well as in vivo or in vitro experiments, the percentage of apoptosis and cell death types can vary. Apoptosis, chondroptosis, necrosis, and autophagic cell death are described in this review. The question of cell death causality in OA progression is also addressed, as well as the molecular pathways leading to cell death in response to the following inducers: Fas, Interleukin-1β (IL-1β), Tumor Necrosis factor-α (TNF-α), leptin, nitric oxide (NO) donors, and mechanical stresses. Furthermore, the protective role of autophagy in chondrocytes is highlighted, as well as its decline during OA progression, enhancing chondrocyte cell death; the transition being mainly controlled by HIF-1α/HIF-2α imbalance. Finally, we have considered whether interfering in chondrocyte apoptosis or promoting autophagy could constitute therapeutic strategies to impede OA progression.

## 1. Osteoarthritis

Osteoarthritis (OA) is the most common chronic joint disease, affecting millions people worldwide. OA incidence increases with age, and approximately 80% of the population displays radiographic evidence of the disease after 65 years of age [1,2]. OA mainly causes pain, stiffness and mobility difficulties, altogether leading to progressive disability and alteration of patient quality of life. Medical care is mainly based on alleviating pain symptom using nonsteroidal anti-inflammatory drugs (NSAIDs), and ultimately ends with joint replacement surgery [3]. Viscosupplementation has also been recommended by the European Society for Clinical and Economic Aspects of Osteoporosis and Osteoarthritis (ESCEO). Intra-articular (IA) injection of hyaluronic acid can reduce pain and increase function notably in the management of knee OA [4]. The etiology of OA is far from being well-known. Its heterogeneity results from a combination of various risk factors and initiating mechanisms [5]. Accidental joint insult (i.e., trauma) can facilitate the development of OA and is, therefore, called post-traumatic arthritis or secondary OA. Primary OA is associated with risk factors, identified and classified as non-modifiable (i.e., genetic and epigenetic factors, age, gender, and ethnicity) and systemic (e.g., obesity) factors.

OA pathophysiology was, for a long time, attributed to biomechanical constrainst exerted on weight-bearing articulations (e.g., knees, hips). However, metabolic factors are also well recognized as mediators in the onset of OA [6]. Adipose tissue can act as an endocrine organ, releasing bioactive molecules, such as pro-inflammatory cytokines (e.g., tumor necrosis factor-α,TNF-α) and adipokines (Resistin [7,8], Visfatin [9], Leptin [10,11,12], and acute-phase serum amyloid a (A-SAA) [13]), of which levels can be significantly and positively correlated with cartilage degradation in OA patients [14]. At the anatomic level, OA is characterized by a progressive breakdown of articular cartilage, involving the remodeling of all joint tissues (bone, synovium, ligaments) with the appearance of osteophytes, synovial inflammation, subchondral bone thickening, and in fine joint space narrowing [1]. Cartilage degradation constitutes one of the prominent hallmarks of the disease.

Articular cartilage is a conjunctive tissue composed of only one cell type, chondrocytes, enclosed in a self-synthetized extracellular matrix (ECM). These specific cells represent approximately 1% of total cartilage volume and are responsible for matrix composition and integrity, thereby conferring to cartilage its functions of mechanical support and joint lubrication [15]. Cartilage ECM is made of water, collagens (essentially type II), aggregating proteoglycans, and hydrophilic macromolecules [16,17]. During OA development, progressive cartilage degeneration is observed. Histochemistry analyses have demonstrated the formation of chondrocytes clusters, the presence of irregular surfaces (fibrillations), cartilage volume loss, and matrix calcification in OA cartilage compared to normal cartilage [18]. These changes in cartilage structure are linked to the alteration of molecular components of ECM. Typically, the decrease in proteoglycan becomes prominent with disease progression and can be evidenced by reduced Safranin-O staining [19]. Distribution of the collagen II network is modified, being uniformly distributed throughout the normal cartilage layers, but at a decreased level in OA-degenerated areas and at an increased level in chondrocytes clusters [18]. Breakdown of these components are handled by a set of aggrecanases (e.g., a disintegrin and metalloproteinase with thrombospondin motifs ADAMTS-4 and -5) and collagenases (e.g., matrix metalloproteases, MMP-1, -3, -8, and -13), which are upregulated, even in early stages of OA-associated cartilage degradation. Pro-inflammatory cytokines, such as IL-1β, TNF-α, and IL-6, are associated with OA occurrence and participate in cartilage degradation through activation of pathways (e.g., nuclear factor-κB_NF-κB, toll like receptor_TLR) that control MMPs and ADAMTS upregulation [20].

Chondrocytes are quiescent cells that rarely divide under physiological conditions: Adult human cartilage is a post-mitotic tissue displaying virtually no cellular turnover [21]. Moreover, the ECM is not innerved nor vascularizated, thereby avoiding new cell supply to compensate for potential cellular loss. As a consequence, phenotypic stability, anabolic/catabolic balance activity, and survival of chondrocytes are crucial for the maintenance of proper articular cartilage. During the course of OA, all of these criteria are modified. Basically, Aigner et al. proposed classifying all chondrocytic changes into five categories: (i) a phase of proliferation (early phase)/cell death (late phase); (ii) disturbed anabolic synthesis activity; (iii) disturbed catabolic activity (chondrocytes that begin to overproduced matrix degradative enzymes); (iv) phenotypic alterations; and (v) osteophyte formation (for an extensive review, see Reference [22]). In the present review, we will focus on chondrocyte cell death and its interplay with cartilage degradation and OA progression. Chondrocyte cell death was reported to occur through apoptosis [23,24], chondroptosis [25], necrosis [26,27], autophagy [28], or a combination of all these processes [26,29].

## 2. Cell Death Mechanisms

Cell death types are classified according to apoptosis (type I, see Section 2.1), autophagic cell death (type II, see Section 2.3), and necrosis (type III, see Section 2.2) [30]. The current view is that these different modes of cell death are not exclusive, but, rather, modulate each other through finely tuned molecular crosstalk [31]. Table 1 lists key features of apoptosis, necrosis, autophagic cell death and chondroptosis, a specific cell death described in chondrocytes (see Section 3.1.5 for more details on chondroptosis).

This table lists key features of apoptosis, necrosis, autophagic cell death as well as chondroptosis, a specific chondrocyte death involving both apoptotic and autophagic components. This Table was established based on informations collected from the following references: [25,32,33,34,35,36].

### 2.1. Apoptosis

Morphological manifestation of apoptosis consists in cell shrinkage (while maintaining membrane integrity), chromatine condensation at the perimeter of the nucleus, membrane blebbing, and, ultimately, formation of apoptotic bodies containing putatively intact organelles [34]. In vivo, these membrane-wrapped vesicles are quickly engulfed by resident phagocytes, thus preventing exposure of intracellular components to the immune system and preventing inflammation [37].

In contrast to passive necrosis, apoptosis is a programmed cell death: At the molecular level, apoptosis consists of sequential scheduled steps, including initiation, effector, degradation, and clearing phases. Apoptosis belongs to normal cell cycle development and is regulated spatio-temporally when cell death is needed at defined locations and precise steps (e.g., interdigital spaces formation, endochondral ossification, ovaries regression) [38]. Apoptosis is also required in order to get rid of infected, damaged, or abnormal cells to avoid any propagation [39]. However, excess or lack of apoptosis can lead to pathological states, such as cancers or autoimmunity, underlying the importance of careful control of this process.

Depending on the stimuli, apoptosis can schematically take two main pathways, the death receptor-mediated pathway (i.e., extrinsic) and the mitochondrial-mediated pathway (i.e., intrinsic) (for an extensive review, see Reference [40]). Although the activation cascades differ, both intrinsic and extrinsic pathway converge toward the activation of executioner caspases (i.e., mainly caspase-3, but also caspase-6 and caspase-7) [41]. Caspase-3 is the most common executioner caspase, responsible for the cleavage of numerous substrates, including cytokeratins, cytoskeletal, and nuclear proteins (including Poly(ADP-Ribose) Polymerase (PARP) and the Inhibitor of Caspase-Activated DNAse (ICAD)), leading in fine to a typical apoptotic dismantling of the cell through DNA fragmentation [42], cytoskeletal reorganization, formation of apoptotic bodies, and phosphatidylserine (PS) exposure on the outer leaflet of the plasma membrane [43], recognized by phagocytic cells for next uptake [44,45,46].

#### 2.1.1. The Death Receptors Pathway (Extrinsic)

The extrinsic pathway is induced by damage or pathogen-associated molecular patterns (DAMPs or PAMPs), and by cytokines activating the TNF superfamily of receptors. Death receptors belong to the TNF superfamily of receptors. All receptors (about 30 different proteins) are type I single passing transmembrane proteins, with the C-terminal part in the cytosol [47]. They are classified into three groups, depending on the conserved domain present in the cytoplasmic tail: The death receptor group includes eight members, known as TNF-receptor 1 (TNF-R1), Fas (also known as DR2, APO-1, or CD95), TNF-related apoptosis-inducing ligand receptor 1 (TRAIL-R1), TRAIL-R2 (DR-5), DR6, ectodysplasin A receptor (EDAR), and nerve growth factor receptor (NGFR) [48].

In the extrinsic pathway of apoptosis, engagement of the death receptor Fas by its ligand FasL leads to the assembly of a typical multi-protein complex called the death inducing signaling complex (DISC). DISC formation allows the recruitment and activation of initiator caspase-8, mediated by the Fas-associated protein with death domain (FADD) adaptor molecule. Recruitment of caspase-8 leads to its autoproteolytic cleavage and subsequent activation [49]. Active fragments of caspase-8 are released in the cytosol where they propagate apoptotic signal by cleaving and generating active effector caspase-3 fragments that will further dismantle cell components [50]. Depending on the amount of active caspase-8 generated by DISC, Fas engagement may require an amplification loop, including Bid cleavage, formation of the apoptosome, and activation of caspase-9 prior to executioner caspase-3 activation [51].

#### 2.1.2. The Mitochondrial Pathway (Intrinsic)

The intrinsic pathway is triggered in response to intracellular damage, cytokine withdrawal, DNA damage, oxidative or endoplasmic reticulum (ER) stress, as well as cytosolic Ca^++^ overload [31]. These stimuli converge in mitochondrial outer membrane permeabilization, which is handled by Bcl2 family members [52]. The activation of its initiator members (BH3-only members, e.g., Bid) both inhibits its pro-survival guardians members (Bcl-2-like members, e.g., Bcl-2, Bcl-X_L_) and allows the oligomerization of pro-apoptotic BH123 members (e.g., Bax, Bak), which thereby disrupt the mitochondrial outer membrane. As a consequence, numerous proteins are directly released from the mitochondrial intermembrane space into the cytosol. Amongst apoptogenic proteins, two classes of proteins can be distinguished, those functioning independently of caspases (i.e., Omi/HtrA2, apoptosis-inducing factor_AIF) [53] and those that activate caspases, either directly or indirectly (e.g., Smac/DIABLO, cytochrome c_cyt c) [54]. Cyt c is the only known molecule able to activate the apoptotic protease-activating factor-1 (APAF-1), a key component of the so-called apoptosome [55]. This multimeric structure is composed of Cyt c-bound oligomerized APAF1, and acts as a platform for recruitment/activation of the typical initiator caspase of the mitochondrial pathway, caspase-9 [56]. In turn, activated caspase-9 can directly process downstream caspases-3/-6/-7, leading to substrate cleavage and execution of apoptosis.

#### 2.1.3. Granzyme B Pathway

Granzyme B (GzB) is one of the most abundant granule serine proteases in mice and humans. It is also the best-characterized protease, having its own proteolytic specificity and some of its substrates have been elucidated [57,58]. Release of GzB is typically encountered in an immune response context where natural killer (NK) and cytotoxic T cells degranulate. The delivery of these cytotoxic proteases (including GzB) inside infected or transformed cells is mediated by the formation of polyperforin-induced pores on cell membranes and ends with cell elimination. Once inside targeted cells, GzB promotes apoptosis via two hierarchies of caspases activation: (i) the direct processing of caspase-3 or (ii) the tBid/apoptosome/caspase-9-dependent cleavage of caspase-3.

In addition to caspases, GzB is able to cleave a wide array of substrates. It contributes to the development of a plethora of diseases, including bone and joint diseases, such as Rheumatoid Arthritis (RA) (reviewed in 2009 by Boivin et al. [58]). GzB specifically cleaves proteins from the ECM, such as aggrecan, fibronectin, vitronectin, and laminin. In human RA chondrocytes, GzB expression was found to be elevated compared to normal chondrocytes, as evidenced by semi-quantitative RT-PCR and immunostaining [59]. Saito et al. also showed that isolated RA chondrocytes displayed NK cell surface antigen, as well as NK cytotoxicity against NK-target K562 cocultured cells. Altogether, Saito et al. proposed that, in RA cartilage, chondrocytes could undergo self-stocked-GzB-mediated apoptosis and display cytotoxicity within affected cartilage [60]. The presence of granzymes A and B in the synovium of OA patients was detected by PCR [61] although very little is known about the role of GzB in OA onset.

### 2.2. Necrosis

Necrosis has been primarily defined as a passive, accidental cell death, caused by injurious stimuli, such as heat, UV radiation, hypoxia, and cytotoxic anticancer drugs. Necrosis is morphologically accompanied by cell swelling (oncosis), mitochondria swelling, cytoplasmic granulation, disruption of the cytoplasmic membrane, and uncontrolled release of cellular contents (i.e., including lysosomal enzymes, DAMPs, as well as a range of pro-inflammatory cytokines) into the intracellular space, thereby exposing them to the immune system and creating an inflammatory reaction [62,63]. Digestion of cell components is caspase- and energy-independent.

However, with time, the concept of regulated necrosis progressively emerged, since specific cytokines, such as TNF-α, induced, in addition to classical apoptosis, morphological changes associated with necrosis, depending on cell types [64]. Further, necrosis can also occur in physiologically relevant situations (e.g., ovulation, immune defense, cellular turnover in the intestine, as well as the death of chondrocytes controlling the longitudinal growth of bones [65]), and in pathological conditions, such as epilepsy or Alzheimer’s disease [62].

Since then, multiple forms of regulated necrosis have been identified, such as necroptosis, parthanatos, ferroptosis, (n)etosis, pyroptosis, and ischemia reperfusion injury (IRI)-mediated necrosis, among which necroptosis is the best-characterized (for further review see Reference [31]). At the molecular level, necroptic cell death results from an interplay between numerous signaling pathways, in which RIP1 kinase seems to be a central initiator [62]. Some authors have also proposed that caspase-independent necroptosis could be induced by pseudoachondroplasia-linked mutation of COMP (D469del-COMP) in chondrocytes [66]. TNF-α was also found to induce rat chondrocyte death, primarily via necroptosis, since necrostatin, but not z-VAD-fmk (caspase inhibitor), were able to reduce propidium iodure (PI) uptake and inhibit TNF-α-induced chondrocyte death [67].

#### Discrimination between Apoptosis and Necrosis

Distinguishing apoptotic from necrotic cell death at the histological level may be difficult, as the two processes can occur independently, sequentially, as well as simultaneously [68]. Moreover, they share common inducers, as well as a common biochemical network, as evidenced by caspase knock-down studies. Indeed, caspase-8 knock-out did not prevent Fas-induced cell death, but, rather, made the cell shift from apoptosis to necrosis, taking an alternate FADD and RIP-dependent pathway, ending with an increased prevalence of necrotic cell morphology [69]. Theoretically, three major commutators can convert an ongoing apoptotic process into a necrotic one: The intensity and duration of the stimulus, the amount of disposable intracellular ATP, and caspases availability [68,70,71].

Various techniques are commonly used to discriminate apoptosis from necrosis. These are based on (i) cytomorphological alterations (transmission electron microscopy_TEM); (ii) cell surface markers of membrane alteration (phosphatidylserine exposure_AnnexinV) vs. membrane permeability ((PI) staining by flow fluorocytometry); (iii) oligonucleosomal DNA fragmentation (DNA laddering in vitro or Terminal deoxynucleotidyl transferase biotin-dUTP Nick End Labeling (TUNEL) allowing DNA strand breaks detection); (iv) caspase-3 activation, cleaved substrates (e.g., PARP), analysis of caspases activator/inhibitors; (v) mitochondrial assays (e.g., mitochondrial membrane potential (δΨm) quantified, for example, by J-aggregated fluorescence [72], Bid cleavage, cyt c release, Bax/Bcl-2 ratio) [40,73].

Taking the work of Grogan and coworkers [74] as an example, Aigner et al. suggested to use multiple approaches to make conclusions regarding cell death type [75], especially in the cartilage field, where apoptosis is a spaciotemporal multistep event [73]. In cell death research, TEM is the most accurate method to identify ultrastructural changes imputable to apoptosis or necrosis (considered as a golden standard). Although this expensive method does not allow large number of sample analyses, it provides two- and three-dimensional images inside cells, allowing to understand biological structure–function relationships at cellular, subcellular, and molecular levels [73,75]. In the case of apoptosis, identification of true apoptotic bodies is achieved when a correlation can be made between morphological appearance using TEM and TUNEL positivity [25]. As the presence of these corpses reflect late phase of apoptosis, other techniques should be used in support.

Combining PS exposure measurements and PI incorporation (the well-known AnnexinV/PI assay) can basically indicate early apoptotic engagement (PS+/PI−), since PS exposure precedes PI incorporation whereas, in necrosis, both events coincide (directly PS+/PI+) [76]. However, based on this method, depending on the time point considered, late apoptosis (PS+/PI+) can be confounded with necrosis, emphasizing the importance of combining several methods of detection, caspase-3 activity measurement, or mitochondrial viability [74].

### 2.3. Autophagic Cell Death

Autophagy describes a cellular process consisting in the degradation of a damaged or superfluous intracellular pool of proteins, carbohydrates, lipids, and organelles. It recycles the resulting breakdown products that are further re-used by cells to generate new macromolecules or to produce energy and maintain viability under nutrient stressing conditions (e.g., serum starvation, etc.). However, excess autophagy can be deleterious and lead to cell death [77]. Three types of autophagy have been described (i.e., macro, micro, and chaperone-mediated), and they differ in the mode of cargo delivery. Amongst them, macro-autophagy is the most extensively studied and will be focused on in this review [78].

The autophagic pathway can be upregulated upon the integration of various signals, such as hypoxia, ER stress, energy balance, cytokines, and growth factors [79,80]. The autophagic process occur in successive steps, each of them controlled by the formation of a specific multiproteic complex [81]. During this process, the cargo (i.e., components targeted for recycling) is progressively surrounded by a double-membrane until it is trapped in a spherical autophagosome. The autophagosome then fuses with the lysosome, resulting in an autolysosome, where the cargo is digested by specific lysosomial proteases [82], and the constituents are released and then re-used for biosynthesis or as sources of energy [80].

About 35 different conserved autophagy-related genes (*Atg*) encode for the proteins involved in the main steps of the macro-autophagy process [78]. Briefly, the initiation step requires the formation of the first complex, Unc-51-like kinase 1/2 (ULK1/2)-ATG13-FIP200-ATG101, activated by mammalian target of rapamycin (mTOR) inhibition upon nutrient starvation [83]. The nucleation step further needs the formation of a second complex, Beclin-1-Atg14-VPS34-VPS15 (also called the class III PI3K complex), which results in the phagophore shaping around the cargo. Typical double-membrane elongation and autophagosome achievement (enclosing the cargo) rely on the formation of two conjugates: (i) ATG5–ATG12 conjugate, which associates with ATG16L and (ii) phosphatidylethanolamine (PE) conjugated to LC3 (the microtubule-associated protein 1 light chain 3), the latter being the major constituent of autophagosomes. LC3 cytosolic form (LC3I) is cleaved by ATG4 and is next conjugated with PE by ATG7 and ATG3. This lipidated LC3 (LC3-II) then associates with newly-forming autophagosome membranes. LC3-II remains on mature autophagosomes until its fusion with lysosomes. The conversion of LC3-I to LC3-II is considered as a specific autophagy marker [84].

## 3. Apoptosis in Chondrocytes and Osteoarthritis (OA) Development

### 3.1. Apoptosis Incidence and Contribution to OA Development

Common situations involve chondrocyte apoptosis, such as the terminal differentiation of hypertrophic chondrocytes from the growth plate (described in bird [85], pig [86], and rabbit [87]), as well as pathological states involving mice and rat [88] or human [89,90,91] cartilage degeneration, such as OA, or the ultra-rare alkaptonuria [92]. Histological data revealed lacunar emptying and reduced cell density within osteoarthritic cartilage, suggesting that cell death could occur during the OA process and even participate in OA onset [93]. Further studies showed that apoptosis effectively occurred in OA cartilage more frequently than in normal cartilage [24,89,90], and that there was a positive correlation between the number of apoptotic chondrocytes and equine [23] or human [91] cartilage degradation/severity of OA. However, the frequency/rate of apoptotic cells observed in situ, mainly by TUNEL staining in human OA cartilage, is highly variable between studies: 6% [90], 19% [89,91], and even 30%–88% [94]. Aigner et al. reached the lowest rate, identifying two lacunaes containing true apoptotic bodies out of several thousand examined in OA cartilage samples (about 0.1%) [95]. The relative contribution of apoptotic cell death in OA pathogenesis is difficult to assess due to several factors:

#### 3.1.1. Experiment Type

The type of technique used in order to state the nature of apoptosis in cell death. Although many studies agree that apoptosis occurs in chondrocytes, both in vivo and in vitro, when using conventional methods to assess DNA fragmentation (TUNEL) or FACS analysis, it is rare to demonstrate the presence of true apoptotic bodies using electron microscopy [25]. Moreover, positivity with a TUNEL assay could encompass necrotic cells [96], meaning that making conclusions about apoptosis with only a TUNEL assay might compromise the conclusions and lead to overestimation of the apoptosis rate [75]. Therefore, TUNEL should be complemented by TEM.

#### 3.1.2. Kinetic Inconsistency

Because the morphological events of apoptosis are fast and would theoretically end with a quick clearance of chondrocytes, a rate of apoptosis that is too high would not be consistent with the slow kinetics associated with the establishment of a chronic disease, such as OA diseases. Therefore, it may be too simple to limit apoptotic chondrocyte cell death as the main pathogenesis mechanism in OA [21,95,97].

#### 3.1.3. Spaciotemporality

The stage of OA disease, as well as the cartilage layers in which apoptosis occur, also seem to be crucial. Indeed, in the early phase of OA development, chondrocytes proliferate, giving rise to OA-typical clusters [98], whereas empty lacunae and hypocellularity are encountered in a later phase [93,99]. Furthermore, as we will see in Section 4, in an early stage of OA in the superficial and middle zones of cartilage, autophagy is triggered to avoid cell death [29,100]. In later stages, both the apoptosis and autophagy processes could be activated, converging on cell death, suggesting that apoptosis, in some circumstances, could occur as a consequence of first line autophagy [29]. Moreover, some studies restrict empty lacunae into the deep zone of cartilage [27,101], whereas apoptotic cells were also detected in the superficial and middle zones [91]. Finally, in some mechanical stress models expected to mimic trauma-induced OA, chondrocyte cell death can also occur through a combination of apoptosis and necrosis [26,102].

#### 3.1.4. Fate of Apoptotic Bodies

The question of clearance of apoptotic bodies was addressed because phagocytes are absent in the avascular cartilage environment. Some have proposed that, having isolated chondrocytes from degenerated rat cartilage, that phagocytosis could be handled by chondrocytes displaying a dedicated phenotype (i.e., type II collagen^+^/CD163^+^), in which phagocytic activity was measured, suggesting a role in the clearance of apoptotic and necrotic cells [103]. Hashimoto and coworkers demonstrated that apoptotic bodies derived from chondrocytes, treated with sodium nitroprusside (SNP) or anti-Fas, contained both nucleotide pyrophosphohydrolase (NTPPH) and alkaline phosphatase activities and could precipitate Ca^++^ [104]. The authors proposed that apoptotic bodies might contribute to OA pathogenesis by increasing cartilage calcification, as is observed with OA cartilage. Apoptotic bodies could also turn on secondary necrosis in the absence of quick phagocytosis [25]. This phagocytic-free environment also gave birth to the concept of chondroptosis.

#### 3.1.5. Chondroptosis

Several teams analyzing cell death in mammalian growth plates in swine [105,106] and rabbits [65], or in articular cartilage [107], observed condensed cells (also called dark chondrocytes) displaying ultra-structural patterns that were distinct from conventional apoptosis. The term chondroptosis was proposed by Roach et al. to describe this type of cell death, occurring in vivo, in hypertrophic chondrocytes (at the vascular front of growth plate) and in dying chondrocytes inside articular cartilage, free of phagocytes [25]. Chondroptosis shares common features with classical apoptosis, such as caspase involvement [108], cell shrinkage, and condensed chromatin contained in the nucleus. DNA is also likely to be cleaved, since chondroptotic cells are positive in a TUNEL staining assay [65]. Nevertheless, appearance of condensed chromatin in the nucleus is distinct: In chondroptosis, chromatin is spread in patches throughout the nucleus, whereas, in apoptosis, chromatin is found at the margins at the nucleus membrane and is aggregated in large masses. Concerning cytoplasmic changes, drastic differences distinguish chondroptosis from apoptosis. Unlike the apoptotic process, where no increase of organelles is observed, chondroptosis cell death leads to the prominent expansion of the rough endoplasmic reticulum (RER) and the Golgi apparatus. In the chondroptic process, elimination of a cell is phagocytosis-independent and consists of self destruction mediated by three phenomena: (i) the increased ER and Golgi membrane create compartments where cytoplasmic components and organelles are digested [35]; (ii) the formation of multiple autophagic vacuoles; and (iii) the extrusion of cellular material into the lacunae space, leaving remnants and vesicular debris, distinct from the apoptotic bodies formed during apoptosis. The final stage of chondroptosis leaves the lacunae empty [25]. More recently, other studies have reported on morphological changes, similar to chondroptosis, notably after trauma-induced cell death in human and porcine discs [109], as well as in other cartilage-degeneration-linked diseases, such as the extremely rare alkaptonuria (AKU) [92].

#### 3.1.6. Cause or Consequence

Numerous studies have reported the decrease number of chondrocytes with aging and OA progression in cartilage [88,110,111], and such hypocellularity was hypothesized to be the consequence of chondrocyte apoptosis [93]. Meanwhile, multiple studies, performed both in animals and humans, established a positive correlation between the severity of cartilage damage and chondrocyte death by apoptosis [23,90,91,93,108,112,113]. However, it is sometimes difficult to delineate if chondrocyte cell death is a cause or a consequence of OA, as was extensively reviewed recently by Zamli et al. [114].

### 3.2. Apoptosis as a Consequence of OA Progression

Studies of α1 integrin and type II collagen KO transgenic mice bring grist to the mill of apoptosis, as a consequence of the loss of matrix interaction between chondrocytes and their pericellular matrix. α1 KO mice spontaneously develop more severe cartilage degradation, which encompasses an increased number of apoptotic chondrocytes compared to wild-type (WT) littermates [115]. Changes in matrix composition, reflected by *COL2* KO transgenic mice studies, also showed a higher chondrocyte apoptotic rate compared to WT littermates. Indeed, thanks to various approaches (TEM, TUNEL, Bcl-2 decrease), Yang et al. observed an increase of chondrocytes with typical apoptotic features in *COL2*^−/−^ mice femoral cartilage compared to WT cartilage, suggesting that matrix-lacking type II collagen could not support the survival of chondrocytes [116]. A similar apoptosis, related to signal loss with the matrix, has been described in other systems, such as in epithelial cells, and was called anoikis [117]. Our laboratory found that integrin α_V_, β_3_, and β_5_ were upregulated during OA chondrocyte dedifferentiation [118]. The α_V_β_3_ complex was also upregulated at the surface of dedifferentiated OA chondrocytes, compared to freshly-isolated OA chondrocytes [118]. Differential integrin expression status might be crucial in sensitizing chondrocytes to cell death induced by the ECM component. Indeed, type II collagen NH2-propeptide (PIIBNP) encompasses RGD motifs and was able to deliver a death signal in cells expressing α_V_β_3_/β_5_ complexes [119]. Moreover, looking at the severity of cartilage damage and apoptotic rates, several studies support that matrix loss precedes chondrocytes apoptosis [23,24,90,91]. Preliminary matrix disruption could also increase its permeability, rendering chondrocytes accessible to apoptosis-inducers, such as nitric oxide (NO) or cytokines secreted by surrounding cells, such as synoviocytes or other chondrocytes. In line with this, several studies have reported that the presence of collagenases predispose chondrocytes to apoptosis [114]. Moreover, in mice, a decline in autophagy-related proteins is observed with age and preceded cell death and structural cartilage damage [120]. However, it is unclear whether cell death or structural damage occurs after a decline in autophagy [120]. Cartilage degradation was also observed as a consequence of autophagy downregulation in immortalized human chondrocytes (TC28a2) and primary human chondrocytes [121].

Zamli et al. addressed the relevant question of causality, determining the temporal sequence between apoptosis and matrix changes, using a guinea pig experimental model. Indeed, these animals are known to spontaneously develop OA with age [110]. Chondrocyte apoptosis was significantly correlated to cartilage damage and hypocellularity, suggesting that chondrocyte apoptosis is a late event of OA [122]. Zamli et al. underlined that, unlike destabilization of the medial meniscus (DMM) mice model of OA, in which apoptosis was observed in the superficial zone (SZ) and middle zone (MZ), chondrocyte apoptosis in guinea pigs was confined to the deep zone of articular cartilage, perhaps reflecting a different mode of OA induction [122]. Zamli and co-workers further demonstrated that bone remodeling, via subchondral bone plate thickening, preceeded chondrocyte apoptosis and cartilage degradation [123].

A recent study analysed the causal relationship between chondrocytes death and cartilage damage using confocal microscopy, standard histology, genetically engineered mice, and DMM surgery [124]. Authors showed that mice whose chondrocytes had been killed (i.e., Diphteria Toxin A (DTA)-ablated) before DMM surgery had significantly less cartilage damage than control mice. Although DMM surgery causes ultimately chondrocytes death, it is the dysfunction of living chondrocytes (i.e., their catabolic phenotype), not their death, that enhances cartilage damage following injury [124].

### 3.3. Apoptosis as a Cause of OA Progression

Chondrocytes apoptosis could also be a pre-requisite for OA establishment, as evoked by other research. In human cartilage, apoptosis, as an initiation step for OA onset, comes from the observation of an increase number of apoptotic chondrocytes [91], or a decrease in the number of chondrocytes [125], or even an apoptosis-related gene perturbation [126] within relatively macroscopically-normal OA cartilage. Similarly, normal *Atg5* conditional KO mice, which develop OA faster than WT mice, also display enhanced chondrocyte apoptosis at two months of development, whereas cartilage does not even display OA-related signs. Bouderlique et al. believe that this early loss of chondrocytes facilitates OA development with age, as the first signs of age-related OA appear only at six months [127]. Moreover, in animal studies, chondrocyte apoptosis in mechanically-compressed bovine cartilage was detected at a lower pressure than that required to stimulate cartilage matrix degradation, suggesting that chondrocyte apoptosis may, therefore, be an earlier response to tissue injury, preceding OA features [128]. Finally, cellular remnants (either apoptotic bodies or remnants from chondroptosis) present in the pericellular matrix or in the chondrocyte lacunae, could eventually release their contents, including proteases, thereby contributing to matrix degradation.

In conclusion, although chondrocyte cell death occurs in OA and is associated with cartilage degradation, it remains unclear if cartilage degradation comes before or after chondrocyte cell death [114].

### 3.4. Chondrocytes Apoptosis in OA Compared with Rheumatoid Arthritis (RA)

Rheumatoid arthritis (RA) is a chronic systemic inflammatory disease that affects multiple joints. Proinflammatory cytokines and autoantibodies induce synovial hyperplasia and cartilage degradation, ending up with progressive destruction of articular structures [129]. RA synovial fibroblasts are characterized by a typical apoptosis resistance resulting in hyperplasia whereas increased frequency of chondrocytes apoptosis is reported in RA [130]. Indeed, Kim et al. compared apoptotic chondrocytes rate in normal vs. RA cartilage samples. They found that apoptotic chondrocytes were more frequently detected in human RA cartilage than in normal (30% vs. 1%) as measured by TUNEL experiment [131]. Using TEM, authors did not find bona fide apoptotic bodies but they observed other features consistent with apoptosis, like cells with chromatin condensation and volume shrinkage. Interestingly, Bcl-2 expression was significantly lower in RA compared to normal cartilage (23% vs. 43%). However, no difference in Fas gene expression between RA and normal cartilage was observed [131].

Interestingly, numerous observations were consistent with higher level of chondrocytes apoptosis in RA. Levels of NO [132] and of two apoptosis related genes (i.e., c-myc and p53) [113] were reported to be increased in RA cartilage. In addition, several substances found abnormally elevated in RA synovial fluid, such as pro-inflammatory cytokines (i.e., TNF-α, IL-1β and IFN-γ) [133], Stromal cell-Derived Factor-1 (SDF-1) [134] or Immune Complexes (IC) [135] were also able to stimulate chondrocyte apoptosis, contributing to cartilage destruction in RA. Recently, Advanced Oxidation Protein Products (AOPPs) have been found accumulated in patients with RA [136]. AOPPs could accelerate cartilage destruction in rabbit arthritis model [136] and could induce chondrocyte apoptosis through the Receptor for Advanced Glycation End products (RAGE)-mediated, redox-dependent intrinsic apoptosis pathway in a rat model [137]. In vitro, AOPPs induced human chondrocyte death through a redox-dependent pathway, including RAGE-mediated, NADPH oxidase-dependent ROS generation, poly (ADP-ribose) polymerase-1 (PARP-1) activation [138] as well as ER-dependent signals [136]. Interestingly, exposure of human chondrocytes to AOPPs significantly increased the production of catabolic factors such as COX-2, MMP-3 and MMP-13, also contributing to RA progression [139]. Finally, osteopontin (OPN) was reported as a deleterious factor in RA. Indeed, OPN induces chondrocytes apoptosis in RA mouse model whereas chondrocyte apoptosis can be suppressed significantly in OPN-deficient mice [140].

Several substances demonstrated an anti-apoptotic function in rat chondrocytes. Indeed, intra-articular injection of osteoprotegerin (i.e., via adenovirus-mediated osteoprotegerin vector (Ad-OPG) reduces proteoglycan loss and prevents chondrocyte apoptosis in a collagen-induced arthritis rat model) [141]. In vitro, amiloride (inhibitor of acid-sensing ion channels) provides protection against acid-induced apoptosis in rat chondrocytes through the restoration of mitochondrial membrane potential and Bcl-2 mRNA level [142]. Interestingly, prolactin (PRL) inhibits apoptosis both in vitro and in vivo [143]. In vitro, PRL inhibits the apoptosis of rat cultured chondrocytes in response to a mixture of proinflammatory cytokines (TNF-α, IL-1β, and IFN-γ) by preventing the induction of p53 and decreasing the Bax/Bcl-2 ratio through a NO-independent, JAK2/STAT3–dependent pathway. In vivo, eliciting hyperprolactinemia in rats before or after inducing the adjuvant model of inflammatory arthritis reduced chondrocyte apoptosis whereas proapoptotic effect of cytokines cocktail was enhanced in PRL receptor–null mice [143].

Recently, autophagy also emerged as a key player in the pathogenesis of RA, affecting mainly synovial fibroblasts, osteoclasts and CD4^+^ T cells function and behavior (for an extensive review, see Reference [144]). By contrast with OA, no information has been reported about autophagy in the chondrocytes of patients with RA [144]. Recently, arthritic hTNFα-transgenic mice transplanted with bone marrow cells lacking Atg7 (i.e., via LysMCre-mediated knockout of Atg7) were protected from TNFα-induced loss of proteoglycan and chondrocytes death, suggesting that autophagy might play a role in both the progression of experimentally induced RA and regulation of chondrocytes apoptosis [145].

Similarities and differences exist between OA and RA chondrocyte apoptosis. Indeed, as observed in OA, increased chondrocytes apoptosis occurs in RA compared to normal cartilage [24,131]. As observed by Aigner et al. in OA cartilage samples, Kim et al. failed to find true apoptotic bodies in RA cartilage slices, suggesting similarities. However, Yatsugi et al. found a significantly greater number of In Situ (IS)NEL-positive chondrocytes in RA cartilage compared with OA cartilage, suggesting that RA chondrocytes are more prone to apoptosis than OA chondrocytes [113]. In rodent, studies showed that the protective properties of osteoprotegerin relied on the blockade of chondrocyte apoptosis in both OA [146] and RA [141] models, suggesting common regulation of OA and RA rodent chondrocytes cell death.

Some key pro-inflammatory cytokines (TNF-α, IL-1β) or mediators (NO) are elevated in both OA and RA serum/synovial fluids compared to normal, that may induce cell death in OA and RA chondrocytes. However, some synovial fluid marker are more specific : For example, Programmed Cell Death PDCD5 levels in RA patients were significantly higher than those in OA and healthy controls and might regulate chondrocytes fate in RA [147].

By contrast to OA, RA is an autoimmune-mediated inflammatory disease. Malemud et al. pointed out the ”apoptosis duality“ occuring in RA. Indeed, the elevated frequency of chondrocyte apoptosis in RA cartilage is opposed to the “apoptosis-resistance” observed in RA synovial tissue, where aberrant survival of activated immune cells, macrophages, monocytes, dendritic cells and synovial fibroblasts is encountered [130]. Therefore, any therapeutic strategy based on the inhibition of chondrocyte apoptosis in RA would lead to a simultaneous aberrant survival of synovial fibroblasts and activated cells of the immune system [130]. One possibility to achieve the dual objective of inhibiting chondrocyte apoptosis while also impairing the aberrant survival of activated immune cells would be to simultaneously inhibit the pro-survival PI3K/Akt/mTOR-signaling pathway [148]. Malemud et al. also emphasized the need for studies assessing the level of chondrocyte apoptosis following administration of experimental drugs or approved therapies for RA (e.g., anti-TNF) in well-validated animal models of RA [130].

Finally, by contrast with OA, no information has been reported about autophagy in the chondrocytes of patients with RA. To our knowledge, only one study suggests that autophagy might play a role in both the progression of experimentally induced RA and regulation of chondrocytes apoptosis [145].

## 4. Autophagy in Chondrocytes and OA Development

Autophagy is an essential component of chondroptosis and, in excess, it can also lead to autophagic cell death in chondrocytes. However, autophagy can play a dual role in chondrocyte fate, as compelling studies have converged on the cytoprotective role of autophagy in OA development [100,121,149,150]. More precisely, it seems that the chondrocyte death process evolves during OA progression and depending on the cartilage layer considered [29]. Chang et al. showed that rapamycin (a potent autophagy inducer) could protect young chondrocytes, whereas excessive activation of autophagy led to autophagic cell death in OA chondrocytes in vitro [28]. Almonte-Becerril et al. demonstrated that during the early stage of OA, autophagy could be activated with the aim of avoiding cell death in the superficial and middle zones, whereas in the late stage, both processes could be activated, converging on cell death, as is described in chondroptosis [29]. In the deep zone, no marker of autophagy were detected, whereas increased active caspase-3 and DNA degradation were observed, suggesting that it is apoptosis that takes place in that zone, which is associated with the abnormal calcification of cartilage observed in OA [29]. Accordingly, autophagy-related proteins ULK1, Beclin-1, and LC3 were highly expressed in human chondrocytes clusters (encountered in the early phases of OA development), whereas a reduced expression of these proteins was observed with age, and were associated with increased apoptosis and decreased cartilage cellularity [100,120,151].

Several researchers strengthened knowledge of the protective role of autophagy against both chondrocyte apoptosis and OA development. In a surgically-induced OA mouse model, Carames et al. associated OA features with a concomitant loss of key regulators of autophagy, and with an increase of apoptosis, as measured by PARP cleavage [120]. Moreover, silencing of beclin 1 resulted in enhanced chondrocyte death [152]. Induction of autophagy (rapamycin-induced) abrogated IL-1β-induced intracellular ROS production, possibly by removing damaged mitochondria, thereby protecting chondrocytes from IL-1β-induced OA-like changes [151]. Recently, Lopez de Figueroa et al. demonstrated that autophagy protected cells from mitochondrial dysfunction and apoptosis, using the mitochondrial stressor oligomycin [150]. Olygomycin induced lower mΔΨ, higher ROS production, higher apoptosis with a lower LC3 puncta (i.e., dots visualized by immunofluorescence), and higher mTOR activation suggesting that autophagy was defective in chondrocytes displaying mitochondrial dysfunction. Restoration of autophagy by rapamycin and torin1 pretreatment impaired oligomycin-induced apoptosis, as well as the decrease of ΔΨ in chondrocytes, and provoked an increase in LC3 puncta [150]. In line with this, treatment with rapamycin reduced the severity of experimental OA [153]. An interdependence was observed between mitochondrial function and autophagy, as Atg5-silenced chondrocytes induced mitochondrial dysfunction (i.e., ROS production and mΔΨ decrease) [150]. In line with this, mice bearing targeted deletion of Atg5 in chondrocytes promoted the development of OA (according to OsteoArthritis Research Society International (OARSI) scores) and an increase of chondrocyte apoptosis rate (as assessed by caspase-3 and -9 cleavage) at two months of age [127]. Accordingly, autophagy could protect articular cartilage from OA, likely by promoting chondrocyte survival. Overexpression of mTOR in OA, resulting from the inhibition of autophagy in the articular cartilage, correlated with increased chondrocyte apoptosis and promoted cartilage degeneration [154]. Vasheghani et al. went further, observing again increased apoptosis and decreased autophagy in PPARγ inducible KO mouse models [155].

### Autophagy Regulators in Chondrocytes: The Hypoxia Inducible Factors (HIFs)

The absence of blood vessels in articular cartilage restricts oxygen supply to passive diffusion from synovial fluid and subchondral bone to chondrocytes [156]. An oxygen gradient was estimated in cartilage, exposing chondrocytes of the joint surface to approximately 6%–10% O_2_ whereas chondrocytes from the deepest layers of the articular cartilage may have access to only 1%–6% O_2_ [157]. Adaptation to such a hypoxic environment is reported to be mediated by hypoxia-inducible transcription factors HIF-1 and -2.

HIF-1 is a transcription factor known as a heterodimer, consisting of two different subunits, HIF-1α and HIF-1β. Under normoxic conditions, the α subunit is degraded, whereas, under hypoxic conditions, HIF-1α degradation is prevented and its accumulation is enhanced. HIF-1α, then, translocates to the nucleus and further associates with its corresponding β subunit to form an active HIF-1 transcription factor [158]. Interestingly, in chondrocytes isolated from normal or OA cartilages, and cultivated in suspension under normoxic conditions, HIF-1α was detected in nuclear extracts whereas dedifferentiated OA chondrocytes did not express HIF-1α. Furthermore, HIF-1α was detected in the nucleus of chondrocytes, in situ, in normal and OA cartilage sections [156].

The importance of HIF-1α for chondrocyte survival was brought about by transgenic studies, revealing that HIF-1α conditional KO resulted in massive chondrocyte cell death (i.e., TUNEL positivity) in the growth plate [159]. The mechanisms of cell protection may involve the modulation of CDK inhibitor p57 expression, as p57 mRNA is absent in the HIF-1α^−/−^ growth plate, and also from vascular endothelial growth factor (VEGF) signaling [159]. In the growth plate, Bohensky et al. showed that, prior to their apoptotic cell death, maturing chondrocytes survive in an autophagic state, stimulated by HIF-1α [152]. The mechanisms of autophagy induction by HIF-1α could involve the modulation of beclin-1/Bcl-2 complex [152], as well as the activation of the AMPK enzyme, resulting in the inhibition of mTOR [160]. Hypoxia- and thapsigardin-induced autophagy share a common HIF-1α-AMPK-mTOR axis of autophagic induction, since AMPK is activated in a HIF-1α-dependent manner in response to thapsigardin in chondrocytes [160]. Recently, Zhang et al. supported the role of Bcl-2 modulation in HIF-1α-mediated autophagy [161]. Hypoxia maintained chondrocyte phenotype (i.e., measured as Sox9 and type II collagen mRNA increase associated with MMP-13 and ADAMTS5 mRNA decrease) and promoted autophagy (i.e., measured by ULK1 and ATG5 mRNA increase). In parallel, hypoxia also increased miR-146a expression in a HIF-1α-dependent manner and miR-146a promoted autophagy by decreasing Bcl-2 expression, an autophagy inhibitor [161].

In contrast, HIF-2α is a potent negative regulator of the autophagic flux [162] and plays a global deleterious role in OA disease. HIF-2α mediates cartilage destruction, as well as chondrocytes cell death, both contributing to the acceleration of OA progression [163]. To control cartilage degradation, HIF-2α can upregulate the expression of matrix degradative enzymes, such as MMP-1, -3, -9, -12, and -13, ADAMTS4, either directly by enhancing their promoter activities or indirectly by cooperating with CCAAT/enhancer-binding protein β (C/EBPβ) [164], or inducing IL-6 [165] or visfatin production [9]. HIF-2α also regulates the expression of other various catabolic factors, such as type X collagen, VEGF, nitric oxide synthase 2 (NOS2), and prostaglandin-endoperoxide synthase 2 (PTGS2), thereby leading to cartilage destruction [166]. As mentioned below, HIF-2α is an apoptogenic factor for chondrocytes via Fas promotion ([167], see Section 5.1.1), but is also a potent repressor of autophagy and HIF-1α expression [162]. The link between HIF-2α and autophagy was primarily made after observing that HIF-2α silencing resulted in the induction of autophagy. Indeed, HIF-2α-silenced chondrocytes (N1511 mouse chondrocytes) displayed concomitant elevation of lysosomal activity, a decrease of mTOR expression, and the presence of autophagosomes (visualized by TEM) [162].

## 5. Cell Death Regulators in Chondrocytes

### 5.1. Inducers

#### 5.1.1. Fas-FasL

From rabbit-cartilage-based studies, Fas levels were reported to be higher in aged cartilage and even correlated with decreased cellularity in articular cartilage during aging [168]. Fas levels were also reported to be elevated in OA compared to normal cartilage [169], whereas other reports showed similar staining in both cartilages [170]. These discrepancies may be due to the clone of Fas antibody used, CH-11 [169] vs. UB2 [170]. Wei et al. and Hashimoto et al. agreed on the Fas distribution in cartilage, was located within the superficial and middle zones, whereas it was absent in the deepest layers.

Analyses of surface expression on chondrocytes isolated from normal and OA human knee cartilages revealed no differences: About 30% of total cell populations expressed the receptor [170]. Agonistic Fas antibody was able to induce typical morphological apoptotic changes in isolated chondrocytes, including in presence of NO synthesis inhibitor L-NMA, suggesting that Fas-induced cell death was independent of NO production [170]. Further insights were obtained for Fas-induced apoptosis, in vitro, with chondrocytes isolated from human normal knee cartilage. Fas-induced apoptosis was shown to rely on caspase-8 but not on caspase-9 activation [171], and Fas/FasL death receptor expression and activity were markedly influenced by chondrocyte density [172]. The activity of Fas was also found to be regulated by its glycosylation level in T cells [173], whereas, to our knowledge, no information is available about Fas glycosylation status in chondrocytes.

In OA-cultured chondrocytes, using simultaneous PI vs. TUNEL or AnnexinV stainings to discriminate necrosis vs. apoptosis, respectively, Wei et al. reported that Fas stimulation could induce both types of cell death [169]. Apoptosis and necrosis could rely on p38, as p38 inhibitor SB203580 abolished Fas-induced cell death, caspase-3 cleavage, and even provoked chondrocyte proliferation. However, Wei et al. did not confirm their observations using electron microscopy-based morphological data. In OA cartilage, authors proposed that Fas stimulation occurred primarily via FasL derived from the neighboring synovium because a significant amount of soluble FasL was found in the synovial fluid of OA patients [174]. As Fas stimulation increased the production of FasL by OA chondrocytes, Wei et al. suggested an amplification loop, facilitating the cell death process, by sustaining and amplifying death signals [169]. Such a simultaneous occurrence of apoptosis and necrosis in an OA context was previously reported in response to OA-mimicking impact damaged canine cartilage when electron microscopy was performed [26].

The Fas/FasL system was also potentiated by other stimuli: HIF-2α [167], 4-hydroxynonenal (HNE) [175], as well as actinomycin D (ActD) [176]. HNE is an end product of lipid peroxydation (LPO). HNE is linked to joint pathology, in particular to OA, because HNE levels are increased in OA synovial fluids, but also because it modulates type II collagen and MMP13 expressions in free radical donors-treated OA chondrocytes [177]. HNE may also contribute to the altering of OA chondrocyte viability by its ability to induce apoptosis, as reflected by caspase-3, -8, and -9-induced cleavage, AIF and Cyt c mitochondrial release, PARP activation, and DNA cleavage [175]. The involvement of Fas/FasL in HNE-induced apoptosis was suggested by two observations: The upregulation of Fas (visualized by WB) in response to HNE stimulation, and the decrease in HNE-induced cell mortality in chondrocytes pretreated with Fas antagonist [175]. Induction of the Fas/FasL system is also one of the main target of hypoxia-induced factor-2α (HIF-2α, encoded by the *Epas1* gene) to induce chondrocyte apoptosis and cartilage destruction in mice models [167]. Indeed, HIF-2α was first described as a deleterious factor in OA because heterozygous mouse *Epas1*^+/−^ suppressed cartilage destruction in a DMM mouse model of OA, whereas overexpression of HIF-2α in chondrocytes displayed spontaneous cartilage destruction [166]. Few years later, it was demonstrated that HIF-2α-induced apoptosis relied on the Fas/FasL activation system. HIF-2α overexpression upregulated Fas mRNA, as well as Fas surface expression, as analyzed in FACS experiments. Moreover, HIF-2α-mediated apoptosis was avoided in Fas-silenced articular chondrocytes, underlining the significance of Fas in HIF-2α-mediated apoptosis. In vivo confirmation was obtained with a Fas-deficient mouse strain, which displayed less chondrocyte apoptosis and less cartilage destruction after OA induction by DMM surgery or Ad-HIF-2α-overexpression [167]. HIF-2α might also facilitate cell death through the inhibition of autophagy [162] (see Section 4). Finally, chondrocyte cell death was increased when anti-Fas was combined with actinomycin D or MG132 [176]. Kim et al. suggested that the sensitization of chondrocytes to Fas treatment was mediated by p53 increase rather than by Bcl-2, Bax, FLICE inhibitory protein (FLIP), or Fas ligand, of which expression levels were unaffected by MG132 or actinomycin D [176].

#### 5.1.2. Tumor Necrosis Factor-α (TNF-α) and Interleukin-1β (IL-1β)

Pathomechanisms of OA, reviewed in 2010, highlighted the role of proteins, such as HIF-2α [166], MMP-13 [178], ADAMTS5 [179], discoidin domain receptor 2 (DDR2) [180], S100A8/9 [181], and Syndecan-4 [182], which, once knocked down, alleviated or even suppressed OA features in an OA mouse model [183]. Overexpression of these proteins, driven by IL-1β and TNF-α in OA (117), led to a progressive loss of collagens and proteoglycans, disturbing cartilage structure and affecting joint stability and function [20]. In addition to the upregulation of degradative enzymes by chondrocytes, IL-1β and TNF-α also prevented the normal production of chondrocyte cartilage matrix components (collagen type II and aggrecan) [184] and stimulated NO production (see Section 5.1.4), thereby contributing, at least indirectly, to cartilage degradation and chondrocyte cell death. In line with this, studies in OA animal models have also demonstrated the beneficial effect of anti-cytokine therapy on chondrocytes [185,186]. However, the interplay and the precise roles of IL-1β and TNF-α in chondrocyte cell death remain conflicting [187].

Indeed, on the one hand, some have stated that IL-1β alone failed to induce apoptosis in chondrocytes [188], and others even attribute a preventative role to IL-1β in Fas-induced apoptosis, relying on NF-κB activation and tyrosine phosphorylation [189]. Using DNA fragmentation and caspase-3 processing techniques, IL-1β was able to enhance apoptosis in 16 out of 22 chondrocyte samples, but not in the remaining six [132]. Some experiments have also showed that cell viability was not modified by TNF-α or IL-1β in cultured chondrocytes, and that ActD was needed to facilitate cell death [190]. The requirement of ActD for TNFα-induced apoptosis was also emphasized by Yoshimura et al. [191]. As described in Section 5.1.4, IL-1β’s capacity to induce apoptosis would rely on co-incubation with reactive oxygen species (ROS) scavengers [192].

On the other hand, IL-1β was described as an apoptogen in rat OA chondrocytes (as evidenced by AnnexinV/PI staining, and caspase-8 and -3 cleavage) [193]. IL-1β could also indirectly induce apoptosis through the upregulation of microRNAs (miRNAs), of which expression was reported in the pathophysiology of various diseases, such as OA [194,195]. Some miRNAs (i.e., miR-34a, miR-146a, miR-139, and miR-98) were involved in chondrocyte apoptosis: They were all overexpressed in OA vs. healthy cartilage and their expressions were induced by IL-1β in OA chondrocytes (miR-34a [196], miR-146a [197], miR-139 [198], miR-98 [199]) or in response to mechanical injury [200]. Concerning the molecular mechanisms of miRNA-induced apoptosis, a recurrent pattern is the repression of anti-apoptotic Bcl-2, as seen with miR-98 [199] and miR-139 [198], and, in some cases, the concomitant upregulation of pro-apoptotic bax [196].

Similarly, TNF-α was also described as a potent inducer of apoptosis [201,202] whereas Yoshimura et al. showed that TNF-α increased Bcl-2 synthesis in chondrocytes, and that cultured cells were resistant to apoptosis using only TNF-α [191]. Our laboratory also previously demonstrated that TNF-α markedly protects human primary chondrocytes from SNP-induced cell death through NF-κB and Cox-2 [203]. Like IL-1β, TNF-α was able to induce apoptosis, but only in combination with actinomycin D, as combined treatment reduced Bcl-2 synthesis and increased apoptosis in a dose- and time-dependent manner [191]. Another study proposed TNF-α as an inducer of chondrocyte cell death through a combination of apoptosis and autophagy. In this model, TNF-α caused the decline of protein kinase CK2 activity, thereby promoting autophagy and facilitating apoptosis [204]. Emphasizing the existing interplay between IL-1β and TNF-α, some studies showed that IL-1β-induced apoptosis could also rely on TNF-α release, observed in rat OA chondrocytes, in response to IL-1β [193].

These puzzling data suggest that the pro-apoptotic or anti-apoptotic effects of IL-1β or TNF-α may depend on the experimental model [190].

#### 5.1.3. Leptin

A high prevalence of OA is observed in obese populations. According to metabolic theory, white adipose tissue release of pro-inflammatory adipokines could participate in OA establishment. Amongst them, leptin has been extensively studied and is considered as a key mediator of OA progression. Leptin is produced, not only by adipocytes, but also by joint cells: Leptin expression was found to be highly expressed in OA cartilage compared to normal cartilage [11]. Our laboratory also described the molecular mechanisms of leptin production in OA synovial fibroblasts [205] and OA chondrocytes [206]. We found that leptin expression is markedly enhanced by prednisolone, is inhibited by genistein, and depends on the activation of Smad1/5 and the glucocorticoid-induced leucine zipper (GILZ) [12,205,207]. Moreover, only dedifferentiated OA chondrocytes were able to produce leptin compared to freshly-isolated primary chondrocytes [206]. Despite the fact that some authors have described an anabolic role for leptin in cartilage homeostasis, leptin was also suggested to participate in cartilage destruction by its ability to induce the expression of multiple MMPs, ADMTS, iNOS, or PGE2 (for an extensive review, see Reference [208]).

Compelling studies have also investigated the role of leptin on the apoptosis of chondrocytes in OA, as well as the underlying mechanisms. In rat articular chondrocytes, leptin induced a ROS-dependent apoptosis, perhaps by increasing lysyl oxidase-like (LOXL3) [209] and decreasing dual specificity protein phosphatase 19 (DUSP19) [210] and uncoupling protein 4 (UCP4) [211]. All these downstream target of leptin were tested in a rat Anterior Cruciate Ligament Transection (ACTL)-induced OA model, and their overexpression (UCP4 [211] and DUSP19 [210]) or silencing (LOXL3 [209]) were found to attenuate/impede OA progression and chondrocyte apoptosis. Interestingly, the silencing of LOXL3, in addition to inhibiting apoptosis, also promoted autophagy [209]. In an in vitro model of OA, application of leptin to chondrocytes provoked ROS and Janus-activated kinase-2 (JAK2)/STAT3-dependent-apoptosis [212]. By contrast, Lee et al. showed that leptin had rather a protective role in vitro against TNF-α/CHX-induced cell death in rat chondrocytes [67]. In line with this, leptin receptor KO mice (*db*/*db*) presented a deficit in postnatal endochondral ossification. *Db*/*db* mice failed to regenerate bone in a fracture-healing model [213]. Mesenchymal and endochondral ossification, normally required to regenerate bone post-fracture, were impaired, probably partly as a consequence of the enhanced and premature apoptosis of chondrocytes [213].

#### 5.1.4. Nitric Oxide (NO) and Reactive Oxygen Species (ROS)

Unlike synoviocytes, chondrocytes display the capacity to produce nitric oxide. NO is a multifunctional molecule that mediates dual role in chondrocytes. NO can be constitutively produced, acting as a second messenger, and plays a critical role in cell proliferation, differentiation, and even in protection against oxidative stress [214,215]. Some experiments have even shown that NO, by itself, is not cytotoxic to cultured chondrocytes [214]. However, an overload of NO can be deleterious, causing cartilage degradation [216] or inhibiting the synthesis of the cartilage matrix [217], provoking mitochondrial dysfunction [72], and even resulting in cell death [72,192].

The link between NO and OA development has been emphasized by several observations. A high level of NO was detected in synovial fluid from arthritis patients [218], and NO synthesis was enhanced in OA cartilage [215]. Moreover, there exists a strong correlation between nitrite production and apoptotic cell prevalence in cartilage harvested during experimentally-induced OA in rabbits [112]. Although NO was spontaneously produced by OA cartilage explants [219], several molecules were found to increase NO production by chondrocytes through inducible nitric oxide synthase (iNOS) upregulation, such as pro-inflammatory molecules (IL-1β, TNF-α, LPS) [220,221] or NO donors [219]. Nevertheless, the role of NO per se in the induction of apoptosis in chondrocytes is still subject to debate and might depend on an upstream NO inducer (IL-1β or SNP).

On the one hand, Blanco and coworkers demonstrated that only NO could induce morphological apoptosis in chondrocytes, whereas ROS (e.g., H_2_O_2_) further oriented cells toward necrosis. Proinflammatory IL-1β, a prototypical inducer of endogenous NO, can also induce ROS production. However, alone, IL-1β failed to induce chondrocytes apoptosis. Nevertheless, when incubated with ROS scavengers, IL-1β was able to drive apoptosis [192]. It could be that IL-1β-induced ROS can react with IL-1β-induced NO, avoiding the biologically active NO to induce apoptosis. Furthermore, co-incubation of IL-1β and inhibitors of NO (e.g., N-Methyl Arginine, NMA) induced necrosis, suggesting that the balance between ROS and NO could orientate the type of cell death [192]. Kim and co-workers also proposed that IL-1β- and TNF-α-induced NO production was responsible for the accumulation of mitochondrial DNA damage, therefore, playing a role in apoptosis of human normal and OA chondrocytes [222]. NO from exogenous and endogenous sources induced apoptotic insults to human chondrocytes via a mitochondria-dependent mechanism [223]. Meanwhile, the systemic administration of iNOS inhibitor, *N*-iminoethyl-l-lysine (L-NIL), in experimentally induced OA in dogs resulted in a reduction of articular cartilage damage as well as a decreased level of cell apoptosis and caspase-3, as determined immunohistochemically [224].

On the other hand, the NO-donor SNP has been largely used to study cell death in cultured articular chondrocytes [72,192,219,225]. Although NO generated downstream SNP was reported to induce apoptosis in these cells, accumulating studies emphasized on the role of ROS-production downstream NO in SNP-induced apoptosis. In this line, Del Carlo et al. concluded that NO-induced cell death required ROS generation to occur in human cultured chondrocytes [214]. In rabbit studies, Liang et al. also proposed that the co-production of ROS with NO could explain the cytotoxicity of SNP, since ROS scavenger resveratrol avoided SNP-induced cell death [226]. Feeding this hypothesis, NOC12, a more potent producer of NO than SNP, was less able to induce cell death compared to SNP [227]. Indeed, NOC12, although able to create mitochondrial dysfunction (i.e., decrease the mδΨ, and reduce intracellular ATP level), failed to form apoptotic bodies in contrast to SNP [227]. In another study analyzing human chondrocytes from various OA grades, SNP-induced cell death displayed features of both apoptosis and necrosis [219]. Caspase-3 was not detected by immunoblotting experiments using 0.2 to 1 mM SNP. SNP induced low levels of internucleosomal DNA fragmentation, as measured by ELISA, whereas it causes extensive, large-scale degradation of nuclear DNA, as observed by a TUNEL assay, independently of p38. This DNA degradation was profoundly inhibited in the presence of NAC, a ROS scavenger, suggesting again the requirement of ROS in SNP-induced cell death [219]. Accordingly, intra-articular injection of antioxidants, such as *N*-acetylcysteine (NAC) or eicosapentaenoic acid (EPA), prevented cartilage destruction and apoptosis, in vivo, in a rat model of experimentally-induced OA [228] or in a DMM mouse model of OA [229], respectively. SNP-induced NO could induce cell death via the downstream production of ROS, NAC, neutralizing NO-induced ROS in rabbit cultured chondrocytes. The apoptosis-preventing activity of NAC was mediated by glutathione, as a glutathione synthetase inhibitor abrogated NAC protective effects [228]. In vitro, EPA and NAC were able to reduce SNP-induced caspase-3 cleavage, as well as p53 [228,229] and p38 phosphorylation [229]. Other studies have supported that NO was not able to induce apoptosis, as the production of high levels of endogenous NO (by adenoviral-mediated over-expression of the *iNOS* gene) in adenoviral transfected chondrocytes failed to induce cell death [230].

The apoptosis signaling events leading to cell death after SNP treatment in chondrocytes usually involves mitochondrial perturbation, such as reduction of mδΨ and Complex IV activity [72], release of cyt c and AIF, cleaved Bid mitochondrial translocation [231], Bax and Bak activation [226], as well as blockage of respiration and ATP generation in human articular chondrocytes and TC28 cells [232] and rabbit chondrocytes [233]. The latter studies suggested that mitochondrial perturbation could contribute to matrix loss and cartilage mineralization, or to the inhibition of cartilage matrix synthesis [232,233]. Interestingly, glucose level seems to also play a role in SNP-induced apoptosis. Indeed, although Maneiro et al. showed that SNP-induced apoptosis via specific inhibition of Complex IV, the inhibition of this complex (by NaN_3_) alone was not able to induce apoptosis unless it was in association with glucose deprivation. Dependence on glucose levels for SNP-induced apoptosis was also underline by de Andres et al., showing that the percentage of cell death after SNP treatment decreased as glucose concentration increased [227]. This dependence on glucose was not observed with another NO donor, NOC12 [227].

In human OA [225] and normal chondrocytes [72], SNP induced an increasing caspase-3 activity [225], or caspase-3 mRNA [72], associated with a reduction in the synthesis of the anti-apoptotic Bcl-2 molecule [72,225]. Using a set of specific inhibitors, Notoya et al. showed that SNP-induced DNA fragmentation and viability were dependent on ERK1/2 and p38 (whereas it was only dependent on p38 in rabbit [234]), caspase-3 and -9, as well as on COX-2 expression. SNP treatment strongly induced COX-2, which, in turn, mediated prostaglandin E_2_ (PGE2) production. PGE2 sensitizes chondrocytes to apoptosis [225], but also mediates the anti-proliferative effect of IL-1-induced NO production in chondrocytes [220], conferring to PGE2 a global inhibitory role in chondrocytes. Our previous work showed that SNP induce apoptosis-like chondrocytes cell death characterized by a high molecular weight DNA fragmentation and annexin V-FITC binding in human primary chondrocytes [203]. The proapoptotic effect of SNP was dependent on NF-κB. We also demonstrated that the NF-κB inhibitor, BAY 11-7085, also induces high molecular weight DNA fragmentation [203] and chondrocyte death [203,235]. BAY 11-7085 induced sustained ERK1/2 activation that was downregulated with 15-Deoxy-Δ-12,14-prostaglandin J2 (15d-PGJ2). Of interest, both MEK/ERK inhibitor UO126 and 15d-PGJ2 protected human primary chondrocytes from BAY 11-7085-induced apoptosis [235].

In cultured rat chondrocytes, SNP-induced apoptosis seems to rely also on a perturbed Bcl-2/Bax ratio, on p38 activation [231,236], as well as on cyt c and AIF release [231]. Interestingly, berberine and cilostazol were able to inhibit SNP-induced apoptosis in vitro and displayed similar beneficial effect in vivo. Indeed, in rat with experimentally-induced OA, intra-articular injection of berberine [236] or oral administration of cilostazol [231] attenuated apoptosis of chondrocytes in vivo, increasing the Bcl-2/Bax ratio, as well as diminishing TUNEL and caspase-3 positive cells in IHC experiments. The protective effect was mediated by AMPK signaling activation and inhibition of p38 MAPK activity [236].

In conclusion, although NO generation was involved in apoptosis induction under certain conditions, it is unlikely that NO per se is the only mediator of cell death upon SNP treatment. Other reactive species might contribute to its cytotoxicity, like the primary by-products of the decomposition of SNP, such as the cyanide aninon or the pentacy-anoferrate complex or the reaction product of NO and superoxide anions (i.e., peroxynitrite) [227]. Peroxynitrite was demonstrated to trigger mitochondrial dysfunction, inhibiting mitochondrial Complexes I, II, and III [72], as well as inducing calpain-dependent and caspase-independent cell death [237].

#### 5.1.5. Mechanical Stresses

Traumatic joint injuries resulting from traffic accidents or sporting injuries constitute a known risk factor for the development of secondary OA [238]. Several in vitro impact models have been developed to study the molecular changes associated to trauma-induced OA: In vitro impact models using instrumented drop tower [239], shear stress [240], or compression [128]. Mechanical stresses applied to human cartilage explants [241] or to cartilaginous endplate chondrocytes [242] or to human growth plate chondrocytes [243] result in chondrocytes apoptosis in vivo and in vitro [242] with the associated loss of glycosaminoglycans, aggrecan, and type II collagen [241,244]. In contrast, hydrostatic pressure was reported to increase matrix macromolecule expression [244]. Moreover, it is also important to consider the intensity of the stress applied, which can induce either cell proliferation or promote cell death [243].

Autophagy induction, post injury, is subject to debate, perhaps due to the animal model and the type of injury applied. Indeed, in a rat model of biomechanically-induced degenerative cartilage of the temporomandibular joint (TMJ), some have proposed that autophagy is induced as an early event post injury, as observed by increased autophagosome detection by TEM, increased levels of beclin1 and LC3, as well as decreased mTOR and MAP4K3 activities [245]. In contrast, in bovine and human cartilage explants subjected to mechanical impact, autophagy was inhibited, and rapamycin treatment of mechanically-injured explants induced the expression of autophagy regulators and prevented cell death and glycosaminoglycans (GAG) release [246].

Several reports propose a combination of necrosis and apoptosis in response to impact-damaged canine cartilage [26]. Using the double staining Propidium Iodure (PI) (to stain dead cells)/Fluorescein Di Acetate (FDA) (to stain living cells) on cartilage explants, whether loaded or not, Chen et al. proposed that necrosis is an early event in impact-damaged cartilage, where apoptosis also occurred. A combination of apoptosis and necrosis also occurred in a cartilage explant model, after blunt impact at a 70% strain [102]. The same authors proposed that, depending on the intensity of trauma, different death types occurred as they observed that a blunt impact above 80% strain resulted in necrosis [102].

With respect to the mechanistic, mechanical stress-induced apoptosis could be mediated by MAPK and the mitochondrial apoptotic pathway in rat [242] and an increase of p38 phosphorylation in chondrocytes isolated from human normal and OA cartilages [247]. Oxidative stress has also been underlined in various mechanical stresses, since antioxidants are able to reduce apoptosis induced by shear stress [248] or abnormal cyclic loading [249]. Similarly, a decrease in antioxidant capacity was evidenced in shear-stressed chondrocytes and contributed to their apoptosis [250]. At the signaling level, shear-induced COX2-inhibited PI3K, which in turn repressed antioxidant response element (ARE)/NF-E2 related factor 2 (Nrf2)-mediated transcriptional response in human chondrocytes [250]. Another study highlighted the role of COX2 as a downstream mediator of shear strain, observing that COX2 inhibitors (celecoxib and indomethacin) could avoid shear-induced PGE2 release and apoptosis [239]. Authors concluded that treatments to prevent OA may include methods of minimizing oxidative damage, whereas some others have suggested that a moderate oxidant exposure could render chondrocytes more resistant to oxidative damage [251]. Shear stress induced chondrocyte apoptosis (i.e., membrane PS exposure and nucleosomal degradation) [252] associated with increased NO release [244,252] and modulation of Bcl-2 in OA chondrocytes [252]. Recently, mechanical injury was also shown to cause apoptosis in human articular chondrocytes through the upregulation of miR-146a, which controls VEGF overexpression and Smad4 downregulation in vitro [200]. Similar VEGF upregulation and Smad4 downregulation was observed in vivo, downstream of miR146a overexpression, in an experimentally-induced OA model [197].

### 5.2. Inhibitory Pathways

#### 5.2.1. Wnt Signaling

Zhu et al. emphasized the protective role of Wnt pathway against chondrocytes apoptosis and articular cartilage destruction. Indeed, the inhibition of β-catenin signaling in mice chondrocytes (i.e., using Col2a1-ICAT–transgenic mice) resulted in a significant increase in articular chondrocyte apoptosis, as identified by TUNEL staining, cleaved caspase 3 and PARP proteins immunostaining [253]. Consistently, Dkk-1 (a well-known inhibitor of Wnt/β-catenin pathway) induced chondrocyte apoptosis in OA joints. Indeed, Dkk-1 expression was correlated with inflammatory cytokine levels (IL-1β and TNF-α), pro-apoptosis mediators (Bad and caspase-3 expressions) and TUNEL staining in human OA articular cartilage specimens [254]. Moreover, Dkk1 anti-sense treatment in ACTL-induced OA rat model abrogated chondrocyte and osteoblast apoptosis as subchondral trabecular bone remodeling in OA. Dkk-1 knockdown also decreased Bax in rat OA knee joint [255]. However, function of Wnt pathway in chondrocyte homeostasis and OA pathogenesis remains controversial. For example, Transcription Factor 4 (TCF4), a downstream mediator of Wnt/β-catenin signaling was found elevated in human OA cartilage compared with healthy cartilage and may contribute to cartilage degeneration in OA [256]. In addition, TCF4 overexpression activated caspase 3/7 and induced human chondrocyte apoptosis (TUNEL measurement) [256].

#### 5.2.2. Bcl-2 Signaling

Karaliotas et al. quantified mRNA expression of Bax and Bcl-2 from human normal and OA cartilage tissue samples. The expression ratio of Bcl-2 /Bax was found to be significantly decreased (2.76‑fold) in the OA group compared with the normal cartilage control group (*p* = 0.022), suggesting apoptosis induction [257]. Consistently, *Bcl-2* gene transfection protected human articular chondrocytes against nitric oxide-induced apoptosis [258]. Conversly, Bcl-2 was decreased after pro-apoptotic stimuli (i.e., cytokine withdrawal and retinoic acid), suggesting that Bcl-2 plays an important role in the maintenance of articular chondrocyte survival [259]. Similarly, repression of Bcl-2 is a recurrent pattern for OA related miRNA-induced apoptosis (see Section 5.1.2). However, Kim et al. showed Bcl-2 expression was significantly elevated in OA lesional tissue as compared to non lesional cartilage [24]. Similarly, Iannone et al. reported that Bcl-2 was elevated in disease cartilage whereas Bax expression was unchanged amongst normal, lesional or non lesional cartilage [260]. These observations might suggest that Bcl-2 could regulate cell behavior independently from its control of apoptosis. Indeed, under cytokine withdrawal, constitutive expression of Bcl-2 maintained aggrecan level whereas Bcl-2 silencing resulted in aggrecan mRNA decrease in rat chondrocyte cell lines [261].

#### 5.2.3. Insulin-Like Growth Factor-1 (IGF-1) Signaling

Insulin-like growth factor-1 (i.e., IGF-1) is an anabolic factor investigated for the treatment of OA, notably in gene therapy clinical trials [262]. IGF-1 prevents apoptosis in chondrocytes [263] through Bcl-2 [264] and MAPK/ERK pathway [265]. A recent study analysed the changes in matrix production and gene expression following Adeno-Associated Virus (AAV)-mediated IGF-1 overexpression in equine chondrocytes [266]. Aggrecan and type II collagen were found significantly induced whereas Bcl-2 transcript was unexpectedly decreased. As Bcl-2 expression vary in function of the chondrocytes developmental stages, authors hypothesized that the decreased of Bcl-2 expression might reflect a change from the late-proliferative and prehypertrophic phases to the resting phase upon transduction [266].

#### 5.2.4. TGF-β Signaling

TGF-β can transduce both ALK5/Smad2/3 or ALK1/Smad1/5/8 signals that have usually opposing effects in cartilage. Indeed, signaling via Smad2/3 blocks chondrocyte terminal differentiation but Smad1/5/8 signaling is strictly required for chondrocyte hypertrophy [267]. Typically, ALK1/ALK5 ratio is increased in aging and OA cartilage [268]. Therefore, understanding the receptor balance in vivo is crucial for the resulting response [268]. TGF-β can play anti-apoptotic role in chondrocytes. Venkatesan et al. monitored effects of rAAV-hTGF-β overexpression in human normal and OA articular chondrocytes in vitro and in situ. TUNEL analysis showed that TGF-β overexpression significantly and durably reduced the percentage of apoptotic cells in OA cartilage compared with transduced control (36-fold decrease, *p* ≤ 0.001), bringing back the levels to those observed in control normal cartilage [269]. The protective role of TGF-β was also demonstrated indirectly. Indeed, miR-146a was identified as a miRNA differentially expressed in OA cartilage [270]. miR-146a specifically inhibits Smad4 and therefore, impaired the TGF-β signaling in cartilage. Jin et al. proposed a pro-apoptotic role for miR-146a mediated by the inhibition of TGF-β pathway [200]. Mechanical pressure injury increased the expression levels of miR-146a and decreased Smad4 level in human chondrocytes. Conversely, the knockdown of miR-146a reduced mechanical pressure-induced apoptosis in chondrocytes and upregulated Smad4 expression [200]. Li et al. described miR-146a as an IL-1β responsive miRNA, overexpressed in an experimentally induced OA rat model and able to induce apoptosis in rat chondrocytes notably through Smad4 inhibition [197].

## 6. Targeting Chondrocyte Apoptosis for OA Treatment

Currently, there is no treatment for a full stop of OA progression. Therefore, preventing, limiting, or delaying chondrocyte cell death, in order to maintain cartilage matrix integrity, might constitute a tempting approach.

Caspase inhibitors (such as zVAD-fmk) are the most studied among all of the apoptosis regulators in OA [97]. Notably, zVAD-fmk was tested in vivo in a rabbit Anterior Cruciate Ligament Transection (ACLT) model of OA with a significant reduction of both chondrocyte apoptosis and cartilage degradation [271]. However, Lotz et al. suggest that this strategy might be restricted to post-traumatic arthritis [272]. Moreover, a tight control of the delivery site of these anti-apoptotic agent should be required (limited to the cartilage injury site) in order to avoid the risk of systemic malignancies [21]. In addition, studies have shown that, in chondrocyte conditional caspase KO, chondrocytes shifted towards necrotic cell death, suggesting that cells trying to avoid apoptosis paved the way for another dying process, such as necrosis, if cells were programmed to die, whatever the way, depending on caspase availability and ATP content [68].

Minimizing oxidative stress and preserving mitochondria integrity could constitute an alternative approach. Antioxidants have demonstrated anti-apoptotic and anti-OA effects in rat and mouse models [228,229]. Promoting autophagy could also indirectly act on removing defective mitochondria and the associated oxidative stress. Moreover, as key autophagic proteins were found to be decreased in aging and OA cartilage, restoring autophagy could be considered to delay OA development. In this sense, rapamycin prevented cell death and GAG loss, as well as a decrease in the expression of ADAMTS5 in a mouse model of OA [153]. Meanwhile, as the long-term use of rapamycin could trigger adverse events, increasing studies should aim at uncovering other molecules and signaling pathways displaying an autophagy modulation function in chondrocytes [273]. Notably, glucosamine was shown to be an effective autophagy activator both in vitro and in vivo [273]. Additionally, the function and signaling associated with regulated in development and DNA damage responses 1 (REDD1), an endogenous mTOR inhibitor, was found to be decreased in aging and OA cartilage. According to these findings, restoration of REDD1 can, not only result in autophagy activation, but also improve mitochondrial function in chondrocytes [274]. Nevertheless, promoting autophagy has to be considered cautiously, since it may also trigger the induction of apoptosis and, therefore, exacerbate OA by accelerating cellular loss [77].

Targeting specific miRNAs may provide promising strategies for the treatment of OA: For example, miR-142-3p constituted an anti-apoptotic molecule in vitro, and its overexpression impeded OA progression in mice in vivo, indicating that miR-142-3p might be a potential molecular target for OA treatment [275]. Conversely, miR-155, an inhibitor of autophagy, was found to be overexpressed in OA: Developing an anti-sense strategy might contribute in restoring autophagy in chondrocytes [276].

Furthermore, preserving subchondral bone integrity could be an alternative strategy, as subchondral bone plate thickening seems to be an early event in OA development, which preceded apoptosis of chondrocytes in a longitudinal animal study [123]. Also, therapies dedicated to the reduction of the catabolic phenotype of chondrocytes may protect against post traumatic OA [124].

A better molecular delineation of apoptotic and autophagic processes may help in designing new therapeutic options for OA treatment.

## 7. Conclusions

In this review, we observed that apoptosis was linked to cartilage degradation and OA development. However, apoptosis might not be the only cell death type encountered, and it is very likely that different cell death types (i.e., apoptosis, necrosis, and chondroptosis) can occur independently, sequentially, and even simultaneously, during OA process. The orientation toward cell death type might depend on multiple parameters, such as the OA stage considered, the cartilage layer, the intensity and duration of stimuli, the animal model studied, the in vivo/in vitro situation, as well as the availability of caspases and ATP.

Although an excess of autophagy can lead to cell death, the current view is that the trigger of autophagy in chondrocytes aims to avoid cell death, especially in the early stages of OA. The decline of autophagy observed during OA progression relies mainly on the HIF-1α/HIF-2α ratio, emphasizing them as major regulators of chondrocyte survival/death, skewing the balance toward autophagy or apoptosis.

**Figure 1:**
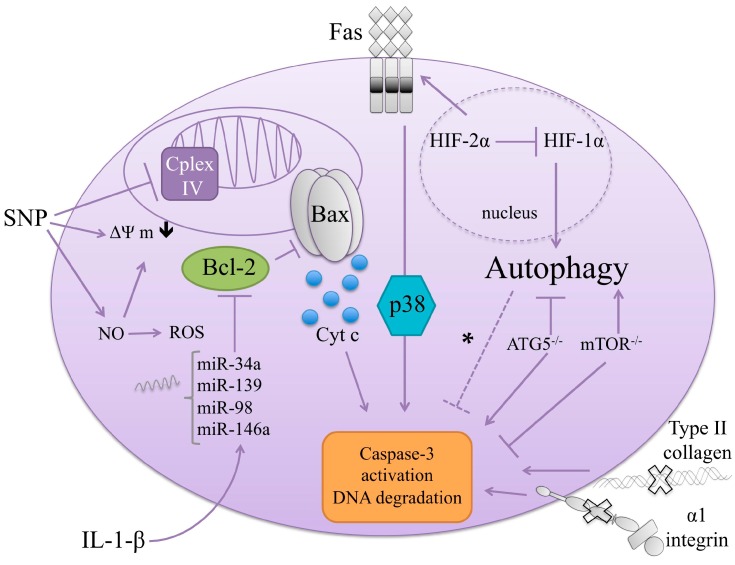
Molecular mechanisms of chondrocyte cell death. Chondrocyte death can be mediated by mitochondria dysfunction, Fas/FasL engagement, as well as by Nitric Oxide (NO) and Reactive Oxygen Species (ROS) production. p38 is often emphasized as a mediator of chondrocyte cell death, using various stimuli. Silencing of type II collagen or α1 integrin can cause chondrocyte cell death. Numerous miRNAs are IL-1β-responsive and play a role in osteoarthritis (OA) development and chondrocyte cell death. * The current view is that the trigger of autophagy in chondroctyes aims to avoid cell death, especially in the early stages of OA. However, the dotted arrow (depicted on the schema) emphasizes that excess autophagy can also lead to cell death.

At the molecular level, chondrocyte cell death in response to Fas, SNP, proinflammatory cytokines, and mechanical constraints delineate recurrent patterns, involving p38 activation, mitochondrial dysfunction, Bcl-2/Bax ratio imbalance, and ROS production (see Figure 1).

## Figures and Tables

**Table 1:** Cell death features. The (*) indicate the exact terms used by Roach et al. [25].

Cell Components/Events	Cell Death Types			
	Apoptosis	Necrosis	Autophagic Cell Death	Chondroptosis
**Chromatine**	Marginal condensation	Fragmented	Absence of condensation	Patchy condensation
**Nucleus**	Fragmentation into apoptotic bodies	Nuclear condensation (pyknosis)	Intact until late stages	Convoluted nucleus Nuclear condensation
**Apoptotic bodies**	Yes	Cell explosion	No	Cellular remnants and vesicules
**Inflammation**	No	Yes	No	Not precised
**ER**	No enlargement	No enlargement	Enlargement	Increase in amount and expansion of lumen (*)
**Golgi apparatus**	No increase	No increase	Enlargement	Increase in early stages
**Autophagic vacuoles**	No	No	Abundant	Frequent
**DNA**	Intranucleosomal cleavage-DNA laddering	Random cleavage DNA Smear	DNA fragmentation occurs very late	Cleaved
**Plasma membrane**	Blebbing but intact	Loss of integrity	Participate to autophagosome formation	Vesicle blebs
**cell**	Shrinkage	Swelling	Shrinkage	Not precised
**Lysosomial enzyme**	Inside apoptotic bodies	Leakage	Inside autophagic vacuoles	Inside cytoplasmic ‘islands’(*) or autophagic Vacuoles
**Elimination (cell fate)**	Phagocytosis of apoptotic bodies	General lysis	Auto-elimination	Auto-elimination of chondrocytes in absence of phagocytes
**Caspases involvement**	Yes	No	No	Yes
**ATP requirement**	Yes	No	Yes	Not precised
